# On Disease of the Omentum

**Published:** 1816-02

**Authors:** 

**Affiliations:** Surgeon


### *On DISEASE of the OMENTUM.*

By Mr. Howship, Surgeon.

THE diseases of the Omentum, at least such as have yet fallen under my own observation, may be said to be few in number. The structure and offices of this membranous expansion are simple. The extended surfaces of the peritoneal laminæ, connected together by a fine intermediate cellular texture, afford convenient space for the termination of innumerable exhalent arteries, breathing forth a genial moisture upon the other viscera of the abdomen, as well as for the occasional deposition of adipose matter.

In several instances I have examined the bodies of the dead, in whom a cancerous affection of the omentum had taken place. In one of these, the disease in the omentum was solitary, but in the other cases, the precise nature of the affection was confirmed, by corresponding disease having taken place in other parts of the body.

In this affection the disease appears to me, to commence in the intermediate cellular texture of the omentum; the healthy action of the capillary arteries is deranged; a morbid secretion is deposited in the interstitial spaces, instead of the fine serous exhalation, while the cellular structure itself, equally with the contents of the cells, undergoes a complete change from health to disease; the texture becomes condensed in proportion as it suffers from distention, it appears fasciculated, and when the disease has made considerable advances, the omentum is found greatly thickened, though in its general dimensions much contracted; the interstitial deposit has produced a congeries of firm, elastic, unequal tumors; the peritoneal covering being thickened, and highly vascular; and when cut into, the disease exhibits the originally fine cellular texture converted into a strong ligamentous fasciculated structure, into the spaces of which the softer contents of the disease are found secreted.

I have in some instances found a variety in the firmness of the general tumor, from the disease being apparently confined to the secretion of interstitial matter, without much correspondent change in the state of the cellular membrane; the matter secreted having the consistence of brain, but without the fasciculated appearance belonging to the above mentioned variety of the disease.

In a late instance, however, I had an opportunity of observing a state of disease in the omentum, which I believe to be very uncommon. The following is an outline of the Case.

A. B. a woman, 76 years of age, who had formerly been healthy, perceived in February 1815, a fulness about the abdomen, which she scarcely noticed, as it gave no uneasiness. It continued, however, to become more and more distinct, although very slowly. In the September following, she was admitted into St. George's Infirmary; the tumor on examination being evidently within the cavity of the abdomen, occupying the situation, and exhibiting very much the appearance of the gravid uterus about the seventh month. The tumor was not painful or tender. It was soft, but still not distinctly fluctuating, though considered to contain a fluid. As it was not believed to be an ovarian disease, it was difficult to tell what to make of it. The general health was unimpaired, and the pulse natural.

The swelling, however, continued to increase. In October the stomach became whimsical and flatulent; symptoms of irritation followed; and the tumor, unless cautiously meddled with, was found to be tender, and somewhat painful.

In the beginning of November, without having previously suffered much pain, or other material change, she sunk, and died.

Examination.—On dividing the parietes of the abdomen, the surface of the omentum was found of a livid grey colour, and lightly adherent to the peritoneum exteriorly. The feel and appearance of the omentum were peculiar. It was not in the least thickened, and yet it formed the immediate parietes of the disease. When carefully separated, I endeavoured to raise up the tender but heavy mass, to see what lay below ; but in the attempt the delicate texture of the fine membrane was ruptured by the weight and quantity of its contents, and several pints of thin sanious fluid ran off, together with several small masses of the grumous substance, which having lost their support, gave way, and separated, by their own weight, from the general mass of the disease.

The subsequent steps of the inquiry demonstrated, that this had been from the first an affection of the omentum alone; the capillary arteries within which, had taken on disease, and instead of secreting adipose matter, had poured out large quantities of the albuminous matter of the blood, and an unhealthy looking serous fluid tinged with the colouring matter. The more solid contents had attached themselves to the sides of the cyst, while the fluid formed the central part of the tumor. The want of cohesion in the disease was such, that it was impossible to save any part, as a preparation.

When cut into, the solid structure of the disease had an equal stain of a grumous red colour, but not from the rupture of a few capillary vessels, the tinge being obviously dependant on the colouring matter of the blood having been secreted together with the albumen.

The disease, in point of consistence, approached most nearly to fungus hæmatodes.

Mill Street, Hanover Square,

Dec. 14, 1815.

